# Captive breeding of pangolins: current status, problems and future prospects

**DOI:** 10.3897/zookeys.507.6970

**Published:** 2015-06-08

**Authors:** Liushuai Hua, Shiping Gong, Fumin Wang, Weiye Li, Yan Ge, Xiaonan Li, Fanghui Hou

**Affiliations:** 1 Guangdong Entomological Institute (South China Institute of Endangered Animals), No. 105, Xin Gang Road West, Guangzhou 510260, China; 2 Guangdong Public Laboratory of Wild Animal Conservation and Utilization, Guangzhou 510260, China; 3 Guangdong Key Laboratory of Integrated Pest Management in Agriculture, Guangzhou 510260, China; 4 Guangdong Provincial Wildlife Rescue Center, Guangzhou 510520, China

**Keywords:** Pangolin, captive breeding, dietary husbandry, disease, conservation

## Abstract

Pangolins are unique placental mammals with eight species existing in the world, which have adapted to a highly specialized diet of ants and termites, and are of significance in the control of forest termite disaster. Besides their ecological value, pangolins are extremely important economic animals with the value as medicine and food. At present, illegal hunting and habitat destruction have drastically decreased the wild population of pangolins, pushing them to the edge of extinction. Captive breeding is an important way to protect these species, but because of pangolin’s specialized behaviors and high dependence on natural ecosystem, there still exist many technical barriers to successful captive breeding programs. In this paper, based on the literatures and our practical experience, we reviewed the status and existing problems in captive breeding of pangolins, including four aspects, the naturalistic habitat, dietary husbandry, reproduction and disease control. Some recommendations are presented for effective captive breeding and protection of pangolins.

## Introduction

Pangolins, also known as scaly anteaters, are eutherians, and unique placental mammals. Despite fulfilling a similar ecological niche as anteaters and armadillos of the order Xenarthra, they are taxonomically distinct. There are eight existing species of pangolins in the world, and all are the class Mammalia, order Pholidota, family Manidae, and genus *Manis*. Pangolins mainly distribute in Eastern, Southeastern and Southern Asia, as well as most of Africa. Four species of pangolins are native to Africa, including the Cape Pangolin (*Manis temmincki*), Giant Pangolin (*Manis gigantean*), Long-tailed or Black-bellied Pangolin (*Manis tetradactyla*) and Tree or African White-bellied Pangolin (*Manis tricuspis*). The other four pangolins are native to Asia, including the Chinese Pangolin (*Manis pentadactyla*), Indian Pangolin (*Manis crassicaudata*), Malayan Pangolin (*Manis javanica*) and Palawan Pangolin (*Manis culionensis*), a new species identified in 2005 (Gaubert and Antunes 2005).

As predators preying on ants and termites, pangolins have a specialized diet and perform an important ecological role in regulating insect populations. It has been estimated that an adult pangolin can consume more than 70 million insects annually, and has a significant impact on the control of forest termites (Shi and Wang 1985). Besides its ecological values, pangolins are extremely important economic animals, valued for both medicine and food. Pangolin’s scales are used as a common ingredient in traditional Asian medicines; for example, the Chinese Pangolin’s scales had been used as an ingredient in traditional Chinese Medicine for thousands of years, and had been recorded in several ancient books of Chinese Medicine. Generally, the pangolin’s scales were thought to have the functions of promoting blood circulation, accelerating milk secretion, detumescence and apocenosis *etc.* But there is no too much progress in determining the scale’s active ingredients. The scales of Indian Pangolin, Malayan Pangolin and Cape Pangolin are also used as medicine by local residents while pangolin’s meat is considered as delicacy and tonic in some Chinese and Vietnamese cultures.

Because of pangolin’s economic values, illegal hunting and illegal trade take place frequently. For example, approximately 24 tons and 14 tons of frozen pangolins were seized in Vietnam and Indonesia in March 2008 and July 2008, respectively (Shepherd 2009); about nine tons and ten tons of frozen pangolins were seized in China and Philippines in December 2012 and April 2013, respectively (GuangzhouDaily 2012; HuanqiuNet 2013). This massive poaching has rapidly exhausted wild pangolin population. Besides illegal hunting, habitat destructions also drastically decreases wild populations (Wu et al. 2002). All extant species of pangolin are listed in Appendix II of CITES (Convention on International Trade in Endangered Species of Wild Fauna and Flora). At the 11^th^ meeting of the Conference of the Parties to CITES in 2000, a zero export quota was established for the Three Asian Pangolin species (Malayan Pangolin, Chinese Pangolin and Indian Pangolin), for specimens removed from the wild and traded for primarily commercial purposes. In 2007, a zero export quota was also established for the Palawan Pangolin following its recognition as a species distinct from the Malayan Pangolin. In 2007, International Union for Conservation of Nature and Natural Resources (IUCN) and Conservation International (CI) adjusted Chinese Pangolin and Malayan Pangolin from low-risk (LR) level to endangered (EN) level (Zhang et al. 2010). In addition, pangolins are also protected in their range states by domestic wildlife laws, for example, Chinese Pangolin is listed in class II national key-protected species in China (Wu et al. 2002).

With the exhaustion of wild populations, captive breeding becomes an important way to protect pangolins from extinction; however, there still exist many technical barriers for the captive breeding programs because of pangolin’s specialized behavior and dependence on natural ecosystems. According to literature, over the past 150 years, more than 100 zoos or organizations have attempted to maintain pangolins. Most captive pangolins died within six months, although some were held for two to three years, a few cases lived for 12–19 years. Zoo records for pangolins in captivity from 1877 to 2001 had been reviewed by Yang (Yang et al. 2007). Currently, a range of pangolin researches and conservation projects are underway both in Asia and Africa, including investigations into illegal trade, ecology, behavior, genetics, rehabilitation, and release. The institutes working on pangolin captive breeding in the last decade were summarized in Table 1. With the accumulation of ecological studies and husbandry techniques, some institutes have made progress in captive breeding programs, and some records of pangolin births have been reported recently. For example, by solving the digestive problems, the Taipei Zoo successfully bred Chinese Pangolins in captivity (Yang et al. 2007); a newborn Malayan Pangolin was reported in the Research Base for Pangolin Domestication and Breeding from South China Normal University in 2011 (SCNU 2011). Although the newborn cases of captive pangolins are encouraging, the captive breeding of pangolins is still difficult, and there is no successful reproduction report of filial generation. More work is needed to improve the livability, growth rate and reproduction rate of the captive pangolins. In this paper, based on the existing literature and our practical experience, we reviewed the status and existing problems in captive breeding of pangolins, and some recommendations are presented for effective captive breeding and protection of pangolins.

**Table 1.** Institutes (programs) holding pangolins in captivity in the last decade.

Institutes/Programs	Species	Status
Taipei Zoo	Chinese pangolin	They improved artificial diet, and had a new-born record in captivity (Yang et al. 2007).
Research Base for Pangolin Domestication and Breeding from South China Normal University, China	Chinese pangolin, Malayan pangolin	The habitat, diet and management of captive pangolins were improved a lot, and had several new-born records in captivity (SCNU 2011).
Yunnan Wild Animals Park, China	Chinese pangolin	Successfully mated pangolins in captivity, and had a newborn record, but the cub died within three days (YunnanNet 2008).
Guangdong Provincial Wildlife Rescue Center; South China Institute of Endangered Animals, China	Malayan pangolin	Supplied pangolins with naturalistic habitats, optimized artificial diet, and kept pangolins for more than 500 days (Programs carried out by ourselves).
Night Safari, Singapore	Malayan pangolin	They can breed and raise the Malayan pangolins in captivity (Vijayan et al. 2008).
Conservation International (CI); Forestry Administration (FA), Cambodia	Malayan pangolin	CI together with the FA have recently set up the Pangolin Rehabilitation Center, to provide care and treatment to animals rescued from the wildlife trade (PangolinSG 2013).
Cuc Phuong National Park, Vietnam; Cat Tien National Park/ Carnivore & Pangolin Conservation Programme (CPCP)	Malayan pangolin	Some pangolins have kept alive over six years, and got two new-born records in captivity; one of the cubs kept alive for nine months (Visited by ourselves in November 2011).
Cu Chi Wildlife Rescue Station, Vietnam	Malayan pangolin	Some pangolins have kept alive over two years, and got two new-born records in captivity (Visited by ourselves in November 2011).
Cambodia/Angkor Centre for Conservation of Biodiversity (ACCB)	Malayan pangolin	ACCB is a pangolin program that has been running since 2004. They have the longest living Malayan pangolin ever to be hand-reared in captivity (SavePangolins 2013).
Indonesian Institute for Sciences (LIPI), Indonesia	Malayan pangolin	LIPI is undertaking research into the rescue and captive-breeding of pangolins in addition to conducting molecular studies (PangolinSG 2013).
Breeding center for pangolins, Nandan Kanan Zoological Park, India	Indian pangolin	The special program is aimed at documenting the behavioral pattern and reproductive characteristic of the Indian pangolin. They had a newborn record at 2007 (ZeeNews 2007).
Tikki Hywood Trust; Zimbabwe Parks; Wildlife Management Authority, Zimbabwe	Cape pangolin	They are working with the Cape pangolin for captive breeding and re-introduction purposes (PangolinSG 2013).
San Diego Zoo, USA	Tree pangolin	One Tree pangolin lives in the San Diego Zoo for visiting (SanDiegoZoo 2013).

## Naturalistic habitat for captive pangolins

Pangolins occupy a variety of habitats in the wild, ranging from tropical to sub-tropical, from cleared and cultivated areas, savannah grasslands to mixed forest, broadleaf forest, secondary forest, *etc.* (Lim 2008b; Wu et al. 2004a), but they have poor adaptability to captive environments. By analyzing their wild habitat and finding the key ecological factors, pangolin’s artificial habitats had been improved a lot. A naturalistic habitat can greatly reduce animal’s stress from changing environments, and keep them comfortable in captivity.

Pangolins are divided into two types: terrestrial and arboreal. Most of the pangolins are terrestrial and they dig burrows or live in other animal’s deserted dens for nesting and shelter. The rest of the pangolins are arboreal, living in hollow trees or on tree branches (Chang 2004). But the distinction between them is not absolute, the terrestrial pangolins also climb tree sometimes. For example, the field study on the ecology of a single female Malayan Pangolin and her young in their natural habitat proved that hollows of large trees were associated with all of their three dens (Lim and Ng 2007). Burrows are extremely important in the terrestrial pangolin’s life history because a suitable burrow could satisfy pangolin’s requirements for food sources, concealment and temperature, but only few studies about pangolin’s burrows were reported (Bao et al. 2013; Jiang et al. 1988; Liu and Xu 1981; Wu et al. 2004b). Chinese Pangolin mainly chooses the soil that is moist, rich and with soft layer thickness to dig burrows. Pangolins’ burrows are blind holes, with no branches. The size of the entrance is generally 14.20 ± 2.79 cm for long diameter, 12.50 ± 2.83 cm for short diameter (Wu et al. 2004b). Interestingly, pangolins’ burrows change with seasons and food resources (Liu and Xu 1981). In summer, the number of burrows is less than in winter, and the burrow is shallower (0.32 ± 0.11 m), including the entrance and tunnel. In winter, the burrow is deeper (1.44 ± 0.73 m). This is probably related to ant’s activities under surface and pangolin’s special requirements for temperature in winter (Wu et al. 2004a; Wu et al. 2004b). In addition, the slope of pangolin’s burrows varies from 30° to 60°, and the openings of burrows often face the sun, probably to make the digging more easily and to maintain the cave temperature in winter. Pangolins’ habitat location is understood to be linked with their key prey species in the wild (Wu et al. 2004a; Wu et al. 2003), and their burrows are always close to ant or termite nests, probably for feeding easily. So a suitable artificial cave or digging condition is believed to be a key factor in the naturalistic habitat for territorial pangolins.

Suitable temperature is another key factor for pangolins’ naturalistic habitat, since pangolins have slower metabolism and little body hair to keep warm. When temperatures were 12–15 °C inside the pangolin cage, pangolins may suffer shivering and a runny nose (Heath and Vanderlip 1988). If they catch a cold, they will be susceptible to pneumonia, which often leads to death (Chang 2004). Determinations of the burrow temperature for the Chinese Pangolin in winter show that the temperature fluctuated between 17.8 °C and 21.0 °C, even the air temperature outside the burrow fluctuated dramatically (during 4.6–38.3 °C). The temperature changes outside the burrow had almost no significant influence on thermal conditions inside the burrow (Bao et al. 2013). Therefore, it was proposed by Bao that the most optimum ambient temperature for Chinese Pangolins in winter is not less than 18 °C.

Most pangolins are nocturnal creatures, occasionally active during the day, and are generally shy and timid (Wu et al. 2004a; Wu et al. 2004b). By analyzing the video records, we found that pangolins prefer to hide in their artificial caves most of the time, except for foraging, suggesting captive pangolins may be always under great stress in captivity. Stress is thought to be one of the major causes of fatality among captive pangolins, and they could be the combined results of the inappropriate environmental factors, including the new surroundings, new diets, too much human interference or handlings, *etc.* Hence, less disturbance, better concealment and reducing pangolin’s stress as much as possible in captive may be other key factors for the naturalistic habitat, and we believe that appropriate burrows or digging conditions in the naturalistic habitat may also be helpful in keeping concealment.

## Dietary husbandry of pangolins

In addition to a suitable accommodation, artificial diet is another critical limiting factor for captive pangolins. Pangolins have adapted to a highly specialized diet of ants and termites making it difficult to replace their natural food completely with artificial food.

Field studies show that except for ants and termites, pangolin’s diet also includes ant larvae, bees (pupas), flies, worms, crickets, and some of other insect larvae, and sometimes sand and grass will be swallowed in the intake process. A pangolin of three kilogram can consume up to 300–400 g of termites per feeding (Coulson 1989; Lim 2008a). Different pangolin species have differences in their diet compositions. Chinese Pangolin’s food includes 15 species, including nine species of termites and six species of ants (Liu and Xu 1981; Shi and Wang 1985; Wu et al. 2005). Cape Pangolin’s food contains more species, including 15 species of ants and five species of termites (Coulson 1989; Jacobsen et al. 1991; Richer et al. 1997; Swart et al. 1999; Sweeney 1956). Diet composition of pangolins is particularly relevant with different latitudes. At the same time, diet composition is also associated with seasonal change. In summer, ants are usually found on ground while termites hide in subsurface tunnels, so ants are the main food source. In winter, ants move into underground nests because of the low temperature, pangolins prefer to choose termites nest for its greater biomass than the ants’. The nutritional components of the ants and termites preyed by pangolins in the wild were analyzed, and the results indicated that the component ratio differs significantly between different ants or termite species. For example, the crude protein, fat, and ash content in three ants species (*Dolichoderus affinis*, *Crematogaster macaoensis*, *Oecophylla smaragdina*) preyed by Chinese Pangolins ranged from 32.65% to 66.85%, 10.85% to 27.26% and 1.91% to 4.81%, respectively (Li et al. 2010). So it is believed that the nutritional differences among their prey could be guidance for pangolins’ food choice. It is interesting that some ants are unacceptable to pangolins, for example, Chinese Pangolin do not like *Paratrechina bourbonica* and *Odontotermes zunyiensis*. An explanation is that except the nutrient components, palatability and safety may also be considered by pangolins.

According to their natural food compositions, a number of artificial diets for captive pangolins had been developed, ingredients including eggs, meat (minced beef, horse meat, fish), milk, milk powders, canned feline diet, orchid leaves, commercial chows, psyllium seeds, carrots, yeasts, multivitamins, and insects *etc.* (Heath and Vanderlip 1988; Tenaza and Schultz 1977; Wilson 1994; Yang et al. 2007). Wu et al. reported that pangolins enjoy high protein, high fat, high calorie food, because of their strong digestion and absorption in their small intestine (Wu et al. 1999). Ke et al. (1999) found that the epidermis of ants have chitin which is suitable for the pangolin’s digestion characteristics. Vijayan et al. believed that Vitamin K is pivotal in treatment of fecal occult blood of pangolins (Vijayan et al. 2008). It is worth mentioning that the Taipei Zoo had made lots of improvements in Chinese Pangolin’s diet by summarizing the previous experiences, and got satisfactory results. The digestive disorders are common diseases for pangolins feeding with artificial food, and the animal’s feces are always fluid. By improving the diet ingredients, the animals’ feces turned from yellow-brown to dark brown and assumed a conical shape. In addition to solving the digestion problems, the diet also significantly improved the survival rate, and they have got a newborn record of Chinese Pangolin in captivity. They also suggested that a certain proportion of the chitin may be the key to pangolin’s artificial diet, this inference agreed with Ke’s study (Ke et al. 1999; Yang et al. 2007), but more studies are needed to further prove it.

Some recipes for captive pangolins were summarized in Table 2, and most of them had been proved effective; they could be good references in pangolin husbandry practice. However, these recipes still need to be improved. Nutrition, palatability, and cost of material should to be fully considered, and in particular, the function of chitin in pangolin’s diet needs to be symmetrically surveyed.

**Table 2.** Some diet formulas fed to pangolins in captivity.

Species	Formulas and remarks
Chinese pangolin	Formula 1: Horsemeat (150 g), milk (180 ml), egg yolk (1), cooked cereal (5 g), milk powder (5 g), calcium powder (1 g), vitamin complex (0.2 ml). Feeding after grinding, mixing, heating. Female pangolin gave birth to a cub. The baby was weaned after 89 days. Both of them survived more than six months (Masui 1967).
	Formula 2: Canned feline diet (2 tin), milk powder (2 tablespoons), flaxseed Meal (2 tablespoons), egg yolk (2). Total feeding quantity of two pairs of pangolin a day. Both of the two female have gave birth, one cub each. A male pangolin survived for 212 days, others more than 460 days (Heath and Vanderlip 1988).
	Formula 3: Bee larvae (100 g), egg yolks (10 g), apple lump (65 g), meal worm larvae (45 g), yeast powder (2.7 g), coconut powder (1.4 g), calcium carbonate (0.9 g), added powdered supplement (1.5 g), vitamin supplements (5 ml), soil (5 g). According to previous formulas, after improvements, the Taipei Zoo developed this formula. Except improved the food intake and digestive disorders, they also got a newborn record in captivity. (Yang et al. 2007).
Malayan pangolin	Formula1: Egg (hard boiled) (2 tablespoons), multi-vitamin liquid (2 tablespoons), horse meat (120 g), water (350 ml), mealworm (150 g), insectivore pellets (80 g), salmon Oil (1 pump), powdered termite mound (4 tablespoons). mixed into a paste, feeding at night (Vijayan et al. 2008).
Indian pangolin	Formula 1: Warm water (1 cup), ant eggs (1/2 cup). There are three pangolins, each had survived for 2, 4, 30 months respectively (Crandall 1964).
	Formula 2: Dry dog food (400 g), horsemeat (200 g), biolac (1/2 tin), raw eggs (2), multiple vitamins (5 drops). A female survived for more than one years, has produced a cub, which only survived for three days (Ogilvie and Bridgwater 1967).
Cape pangolin	Formula1: Minced meat (1/2 pound), maize paste (1/2 pound), raw eggs (2), milk (2 pint), termites (2 pound). Stir the food without termites, and feeding with termites at 11 am and feeding feed at eight pm. The first pair survived for 28 months, and gave birth to one cub. The cub survived for ten months; The other pair survived for 38 months with one cub born. (van Ee 1966).
Giant pangolin	Formula 1: Chopped bovine heart meat (2 cup), cooked cereal milk powder (2 cup). Feeding in the dusk, add raw egg and wheat germ, sometimes. One survived for four years, the other two survived for two years (Crandall 1964).
Tree pangolin	Formula1: Minced beef. Without water, adding vitamin, mixing into wetting powder. A female pangolin lived for two years, gave birth to a cub which lived for seven months (Menzies 1963; 1966).

## Reproduction study of captive pangolins

Reproductive disturbance is another technical barrier for captive pangolins. Only a few newborn records were reported in the last decade. This is an understandable situation, because without a successful diet, even survival and growth are problems for captive pangolins, not to mention successful reproduction. Except for poor adaptation to captive environments, poor understanding of pangolin’s reproductive biology is another limiting factor in pangolin’s captive breeding. In the past, the understanding of pangolin reproductive biology has been limited, owing to fragmentary reproductive records, which are primarily from interviewing with hunters, birthing records of rescued individuals in shelters, and a handful of dead fetus anatomy records. So, the reproduction parameters of pangolins are quite inaccurate. At present only few reproductive traits, including pangolin’s time of birth, estrus, gestation period and litter size were reported (Chin et al. 2011).

Typically, pangolins are solitary except in mating season. May to July is their preferred mating seasons. Male pangolins often fight each other for females in the mating seasons. The winner will mate with the female pangolin, with the mating period generally lasting three to five days. Female pangolins have two to five estrous cycles during the mating season, and each will last for 11-26 days, until pregnancy (Cen et al. 2010). Different species of pangolin have different gestation periods, Cape Pangolin’s gestation period is about 139 days (van Ee 1966), the gestation periods of the Tree Pangolin and Long-tail Pangolin are close to the Cape Pangolin’s; the Indian Pangolin’s gestation period is shorter, just 65–70 days (Hayssen and Van Tienhoven 1993). The pregnancy period of the Chinese Pangolin is about 101–169 days (Wu 1998b; Yang et al. 2007), but by monitoring the concentration of the serum progesterone, Chin et al. believed that the gestation period of the Chinese Pangolin was 318 to 372 days (Chin et al. 2011), which is significantly different from the results of Wu’s and Yang’s observations (Wu 1998b; Yang et al. 2007), suggesting that more systematic and quantitative analysis are needed to determine pangolin’s reproduction parameters. Pangolins are believed to give birth to one young each time, but twins are known (Lim 2008a). The cubs will stay with their mother for six months before they leave. There are suggestions that new-born pangolins reach sexual maturity in one or two years, though there is no consensus on this.

Generally, regarding the pangolin’s reproduction, little information has been known. This situation greatly limits the application of artificial reproductive technology in pangolin’s artificial propagation, suggesting more work is needed to overcome the pangolin’s reproductive disturbance in captivity.

## Disease control for captive pangolins

Because of their poor adaptability to captive environment and a weak immune system, pangolins are easily to get sick. Gastrointestinal disease, pneumonia, skin disease, parasites, *etc.* are the most common causes of death for captive pangolins (Cen et al. 2010; Chang 2004; Clark et al. 2008). Over 50 percent of captive pangolins’ died of hemorrhagic gastric ulcers and pneumonia (Chin and Yang 2008). It is believed that the directing causes of the gastric ulcers are the stress and unsuccessful artificial diet. Under stress, pangolins are easy to develop stomach ulcers and die (Chin and Yang 2008; Chin et al. 2006). To provide an appropriate habitat and reduce the stress may be helpful to improve the health of captive pangolins.

**Figure 1.**
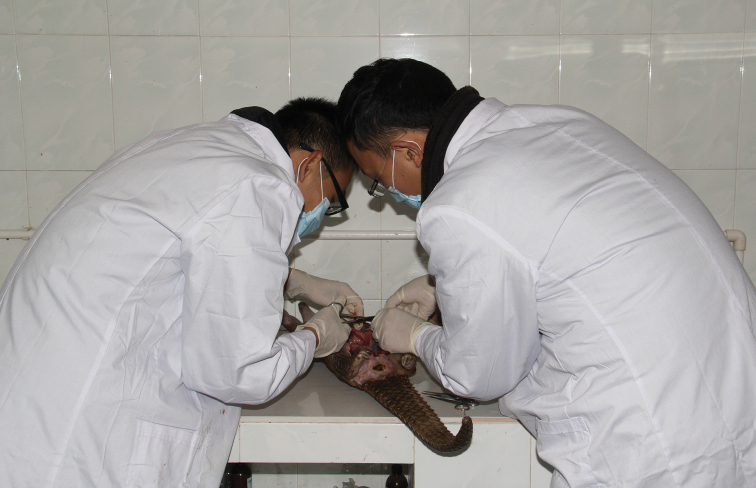
Anatomical study of the pangolin.

It is documented that almost all the wild animals have parasites, such as ticks and mites, especially for the pangolins seized from illegal trade (Hafiz et al. 2012). Due to the mess and dirty transportation process, most of the individuals have had high parasite burdens, and a very high percentage of animals shed gastrointestinal worm eggs in their feces (Clark et al. 2008). According to the autopsy results of 17 Malayan Pangolin seized from the illegal marketing (10 females, 7 males) in December 2013 (Figure 1, anatomical study of the pangolin), 16 individuals carried ticks with an average intensity of 9 and ranged from 1–24 (unpublished data). These parasites probably cause pangolins anemic, wasting, infectious disease, loss of immunity, and even death. Unfortunately, because of lacking of animal samples we can still not conduct detailed research on pangolins’ diseases.

## Lessons from our practical experiences

**Figure 2.**
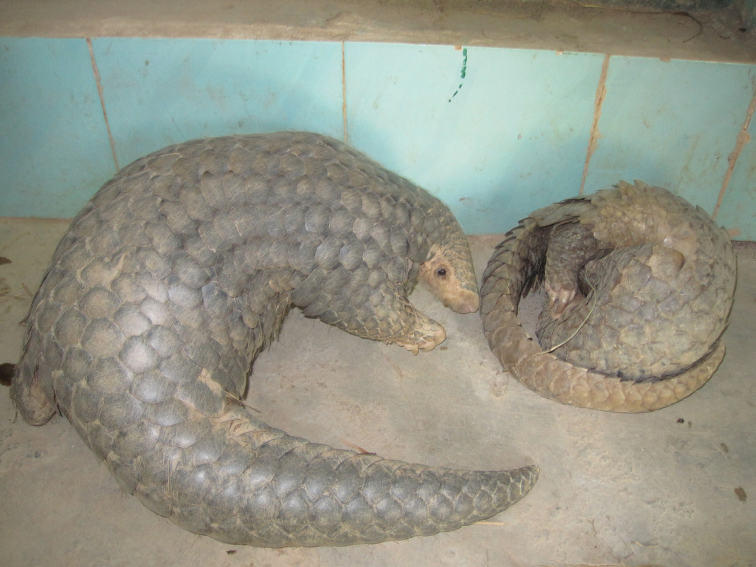
Pangolins in captivity.

Since 2010, in order to protect the seized pangolins, a plan of *ex* situ conservation with captive-breeding has been carried out by South China Institute of Endangered Animals and Guangdong Provincial Wildlife Rescue Center (Guangzhou, China). From 2010–2013, we had kept 35 seized pangolins (2 Chinese Pangolins, 33 Malayan Pangolins) (Figure 2, pangolins in captivity). Based on field survey of the habitats of the both pangolin species, we created an artificial habitat for these pangolins. This artificial habitat is created in a room including caves for resting and breeding, feeding area with food bowls, and play area with dry tree trunks. In winter, the caves are covered by dry straw and cotton quilt to keep the temperature stability. Air conditioners are also used to adjust the temperature. Every day we offer pangolins food (milk and suckling pig feed as major food, and termite as supplementary feed) and clear water and observe the food consumption. A video monitoring system is used to record the activities of pangolins. With this method, although most of pangolins have died of gastrointestinal disease and other unknown diseases, we had kept 2 pangolins (1 Chinese Pangolin, 1 Malayan Pangolin) for over 600 days, and 3 Malayan Pangolins for about 380 days, 12 Malayan Pangolins for about 200 days.

Based on three years of work, we got some practical experiences and lessons: (1) Keep the temperature stable in the pangolin's rooms. The sharp change of temperature has serious effect on pangolins’ health. For a case in 2012, when the cold came suddenly, the temperature dropped from 20 °C to 5 °C in one night, such temperature change caused 6 Malayan Pangolins death within 15 hours. (2) Different species, different sources and different body-sized individuals should be kept in deferent rooms to avoid fighting and to minimize the propagation of disease.

Based on the review of current knowledge and our own experience, we give some recommendations for the future practice of captive pangolins, wishing to improve the successful rate of captive breeding of pangolins.

1) Optimize and quantify the environmental parameters. Naturalistic habitat which could simulate their wild microenvironment is the best choice for captive pangolins. It seems that we have achieved some progress in constructing pangolin’s artificial habitat, for example, moderate temperatures, less disturbance, better concealment, supplying an artificial cave or a mound for digging burrows *etc.* Nevertheless, the detail parameters of the habitat need to be quantified and further optimized in the future, such as the indoor temperature and humidity, area, animal density, light intensity, litter type and height *etc.* Moreover, reducing the stress level of pangolins in captivity must be noted, and installing a closed-circuit television in their habitat will be convenient to observe their activities without interference.

2) Analyze pangolin’s digestive system and improve their artificial diets. Appropriate artificial diet is another key factor for captive breeding of pangolins. Although there are lots of diet formulas developed, and some of them seem successful, more work is needed to improve their artificial diet in the future, because even the most successful artificial diet can’t replace natural food completely. Generally, artificial diet must match their digestive system and satisfy their nutritional needs. This requires us to understand the physiology of pangolin’s digestive system comprehensively. There are some anatomical analysis of pangolin’s digestive system recently (Adeniyi et al. 2012; Munyala et al. 2011; Ofusori et al. 2008), but it is still unknown why pangolin selectively preys on ants and termites. Analyzing pangolin’s digestive enzyme composition and identifying its critical categories may provide us some clues. Further analyzing the nutrition components of the ants and termites preyed by pangolins may help us to understand their nutritional needs. It is particularly worth mentioning that finding the function of the chitin in pangolin’s food is an interesting topic, whether it is the crucial element for pangolin’s nutrition requirements needs more studies. The information about pangolin’s digestive enzyme components and food components may contribute to the further improvement of their diet formulas.

3) Determine the reproductive parameters. Although there are successful breeding records of captive pangolins in the past, they are just individual cases. Improving the reproduction rate is the ultimate aim of the pangolin captive programs, so more reproductive parameters need to be determined in the future. Besides the descriptions for the reproductive traits, such as estrus cycle, gonad activity cycle, mating time, pregnancy period *etc.* Quantitative analysis of hormone concentrations during the reproductive cycle needs more attentions, including testosterone concentration and male sexual behavior, estrogen concentration and estrus, follicle-stimulating hormone (FSH) concentration and ovulation, *etc.* These studies could provide reference for artificial regulation of reproductions in practice.

4) Disease control. Prevention is much more efficient than treatment in disease control. Supplying a suitable environment or using preventive actions to reduce the chance of illness will be the preferred choices for captive pangolins. For example, suitable temperature and humidity is essential to prevent pneumonia, and appropriate artificial diet is essential to prevent gastrointestinal diseases. Pesticides such as thiabendazole can be used regularly to get rid of parasites. At the same time, more case studies need to be strengthened to find the prevalence of disease in pangolins, and to provide references for future treatments.
